# Prognostic Significance of Tripartite Motif Containing 16 Expression in Patients with Gastric Cancer

**DOI:** 10.31557/APJCP.2021.22.8.2445

**Published:** 2021-08

**Authors:** Jalil Afshar, Jamshid Mehrzad, Hassan Mehrad-Majd, Ladan Goshayeshi, Jafar Saeidi

**Affiliations:** 1 *Department of Biochemistry, Neyshabur Branch, Islamic Azad University, Neyshabur, Iran. *; 2 *Cancer Molecular Pathology Research Center, Mashhad University of Medical Sciences, Mashhad, Iran. *; 3 *Department of Gastroenterology and Hepatology, Faculty of Medicine, Mashhad University of Medical Sciences, Mashhad, Iran. *; 4 *Department of Physiology, School of Basic Science, Neyshabur Branch, Islamic Azad University, Neyshabur, Iran. *

**Keywords:** TRIM16, tripartite motif containing 16, gastric cancer, overall survival

## Abstract

**Background and Aim:**

Tripartite Motif Containing 16 (TRIM16) is a member of the TRIM protein family which is known to play a suppressor role in development of numerous tumor types. However, a positive correlation between TRIM16 expression and gastric cancer (GC) progress has created a controversial situation that need to be fully delineated. The aim of this study was to assess the expression level of TRIM16 mRNA and its relationship with β-catenin, CyclinD, and BCL2 expression in Iranian GC patients and to investigate its possible association with patients’ overall survival.

**Materials and Methods:**

The expression level of TRIM16 of fresh primary tumor and adjacent normal tissues in 40 GC patients was evaluated by real-time quantitative PCR method. Moreover, patients were subdivided into high or low expression subgroups based on the TRIM16 expression levels. The relationship between TRIM16 expression level, β-catenin, Cyclin D, BCL2, some clinicopathological data and prognosis of GC patients was also analyzed.

**Results:**

qPCR analysis showed a lower level of TRIM16 in GC tissues (fold change=0.351) in comparison to their matched adjacent noncancerous tissues (P<0.001). Contrary to this, the expression levels of β-catenin, Cyclin D, and BCL2 genes were up-regulated in cancerous samples. This may explain the tumor suppressive function of TRIM16 in GC; as reduction in TRIM16 expression leads to the accumulation of mRNAs from β-catenin, Cyclin D, and BCL2 genes and eventually cancer progression. We did not observe any significant correlation between TRIM16 expression and patients’ overall survival. Univariate Cox regression analysis indicated that anemia, weight loss, bleeding, stomach ache, and smoking are statistically associated with overall survival; while, multivariate analysis did not support any correlation.

**Conclusions:**

In sum, this study suggests a tumor suppressive role for TRIM16 in gastric cancer and proposes it as a potential candidate for GC prognosis.

## Introduction

Gastric cancer (GS; stomach cancer) is the 3^rd^ cause of cancer deaths (after lung and colorectal cancers with 783,000 deaths per year) and the 5th top ranked malignancy in the world annual incidence rates of cancers (about 1 million new cases each year) (Bray et al., 2018). In Iran, and some other Western Asian countries, GC is the prevalent diagnosed cancer and results in the main cancer related deaths between men. The overall 5-year relative survival rate for GC is estimated 20% worldwide; however, remarkable regional variations have been reported (Karimi et al., 2014). Although the Helicobacter pylori is considered as the primary cause of GC, there are several important risk factors (such as diet, lifestyle, environment, genetics, gender, age) on the table (Plummer et al., 2015; Yusefi et al., 2018; Poorolajal et al., 2020).

Unfortunately, GC is mostly identifiable at advanced stages that adversely affect the disease management procedures. Thus, there is an urgent need for early detection of gastric cancer. Biomarkers are potential valuable tools for on time diagnosis and prognosis of cancer, precise therapy, and maximizing the overall survival and patients’ quality of lives (Mehrad-Majd et al., 2019a; Mehrad-Majd et al., 2019b; Mohammadi et al., 2019; Arjmand et al., 2020; Mohammadi et al., 2020). Up to now, extensive research has introduced various biomarkers with different applications and efficiencies in GC (Ye et al., 2020). Yet, the identification of novel and effective biomarkers for accurate and early diagnosis and prognosis of GC is at the heart of efforts and is a major area of interest within the field. Recent breakthroughs in genomics have emerged as a powerful platform to introduce precise nucleotide biomarkers, in addition to proteins, for GC. Moreover, studying the underlying genetic susceptibilities and ethnic background for GC formation and development should be considered (Kim et al., 2014).

The tripartite motif (TRIM) family proteins are encoded by TRIM genes and are known for having a conserved motif (RING Finger-B Box-Coiled Coil (RBCC)) at their N-terminus (Sardiello et al., 2008). The TRIM family is linked with some human diseases including cancer. Some members of this family are associated with tumor development and progression via chromosomal rearrangements, ubiquitination, or loss of anti-oncogenic functions (Cambiaghi et al., 2012). Tripartite Motif 16 (TRIM16), also known as estrogen-responsive B-box protein (EBBP), has been identified in human mammary epithelial cells and contains two B-box domains and a coiled-coil region without the RING domain (Kim et al., 2013). This protein is encoded by TRIM16 gene on chromosome 17p11.2 and some previous research has attributed the tumor suppressive role to it.

Studies have shown that TRIM16 plays a significant role in the retinoid signaling pathway (Cheung et al., 2006). In fact, retinoic acid can cause cell death through its receptor (retinoic acid receptor β (RARβ)) on cancerous cells. Tumor cells counteract this effect by down regulation of RARβ gene expression. TRIM16 acts against this escape mechanism by inducing the expression of retinoid target genes, RARβ and CYP26A1, in retinoid-resistant cancerous cells (Raif et al., 2009). Overexpression of TRIM16 has been shown to decrease neuroblastoma cell growth, cell motility, and tumourigenicity (Marshall et al., 2010). TRIM16 was suggested as a candidate for targeted therapy in cancers expressing high levels of vimentin and E2F1 proteins. The expression level of TRIM16 in prostate cancer tissues was shown to be lower than normal tissues and was directly linked with patients’ overall survival (Qi et al., 2016). It has been confirmed that TRIM16 overexpression inhibits epithelial-to-mesenchymal transition (EMT) process and the invasion of tumor cells in prostate carcinoma and also in non-small cell lung cancer (NSCLC) cells (Huo et al., 2015; Qi et al., 2016). Moreover, high levels of TRIM16 has been demonstrated to induce apoptosis in human breast cancer and neuroblastoma cells through the induction of caspase-2 activity (Kim et al., 2013). To date, few studies have investigated the association between TRIM16 expression and gastric cancer. Yan et al. studied the differentially expressed long non-coding RNAs (lncRNA) and mRNAs in GC and proved the overexpression of TRIM16 in stage IV of GC tissues and in distant metastasis sites (Yan et al., 2015). Although most of the previous studies have indicated down regulation of TRIM16 in other cancers, they found a positive correlation between TRIM16 expression and GC progress. Therefore, considering these controversial results, the role of TRIM16 and its functions in GC are yet to be fully delineated. 

In this study we evaluated the expression level of TRIM16 in Iranian GC patients and its role in patients’ overall survival. Also, the correlation between TRIM16 expression levels and several clinicopathological variables of GC patients was analyzed. Additionally, to shed light on the functions of other biological pathways in GC (carcinogenesis, cell cycle, apoptosis and Wnt signaling), we determined the expression of their representative genes; β-catenin, CyclinD and BCL2. 

## Materials and Methods

*Patients and tissues*

A total of 40 GC patients were recruited in the first affiliated hospital of Mashhad University of Medical Sciences, Mashhad, Iran, between November 2015 and February 2018. Patient selection was carried out by considering four main criteria; I) subjects with GC relevant clinical symptoms such as dysphagia, dyspepsia, gastrointestinal bleeding, anemia, weight loss, anorexia, inappetence, nausea, reflux, and vomiting; II) no history of any treatment for GC including chemotherapy, radiotherapy, and surgery; III) no history of any medications for stomach disorders; and IV) GC verification by pathology tests. Using endoscopy, the cancerous tissues and their corresponding non-cancerous tissues (located at 5cm away from tumor sites) were obtained from. The total average age of patients (31 male and 9 female) was 65.73±10.19. Tissues were kept in RNAlater for RNA preservation (Thermo Fisher Scientific, Waltham, MA, USA) at 4°C overnight and then stored at -80°C until RNA extraction.

*Isolation of total RNA and qPCR*

Totally, 80 tissue samples (40 cancerous and 40 non-cancerous control tissues) were analyzed in this study. Samples were weighed and the total RNA was extracted by Wizol™ Reagent (Wizbiosolutions Inc., Korea) according to the manufacturer’s protocols. NanoDrop 2000 spectrophotometer (Thermo Fisher Scientific, Waltham, MA, USA) was applied to measure RNA concentration at 260 nm. The quality of extracted RNAs was confirmed through agarose gel electrophoresis. 20 μl of WizScript™ RT Master kit (wizbiosolutions, Seongnam, Gyeonggi, Korea) and 5 μl of total RNA were used to synthesize the first-strand cDNA. WizScript™ RT Master kit contained MMLV reverse transcriptase, oligo dT, random hexamer, RNase-free water, RNase inhibitor, stabilizer, Mgcl2, dNTPs, DTT. The PCR parameters included denaturation at 95°C for 5 min, then 40 cycles at 95°C for 15 s, annealing at 57°C for 30 s, and elongation at 72°C for 30 s. Agarose gel electrophoresis was used to examine the quality of synthesized cDNAs. In the following step, the relative mRNA expression levels for TRIM16, β-catenin, Cyclin D, BCL2, and β-actin were measured by qPCR. A Roche PCR machine, SYBR green (AMPLIQON), as described in manufacturer’s instructions, and primers’ sequences (Table 1) were applied for this purpose. The real time PCR protocol was as following: preincubation (94°C for 600s), 40 cycles of amplification (95°C for 15s, 59°C for 30s and 72°C for 30s), followed by dissociation stage (95°C for 10s, 65°C for 60s and 97°C for 1s). All of the experiments were repeated 3 times, independently. The comparative CT method (2^-ΔΔCT^) was considered to calculate the expression levels of TRIM16, β-catenin, Cyclin D, and BCL2 against β-actin (Livak and Schmittgen, 2001). 

*Statistical analysis*

All of the statistical analyses were performed by IBM SPSS Statistics 22 and P-value<0.05 was considered as statistically significant. The paired-samples t-test was used to compare the mRNA expression levels of TRIM16, β-catenin, Cyclin D, and BCL2 between GC tissues and their matched noncancerous tissues. Patients were grouped into two categories of high and low expression of TRIM16. The correlation between TRIM16 expression levels (high and low) and clinicopathological parameters in GC patients were studied using the independent t-test, Chi-square test, and Fisher’s exact test. Kaplan–Meier analysis and Log Rank (Mantel-Cox) tests were applied for overall survival analysis in different levels of TRIM16 expression (Bland and Altman, 2004; Rich et al., 2010). At the end, the effects of clinicopathological variables and TRIM16 expression on overall survival was tested through univariate and multivariate analyses, Cox (proportional hazards) model. 

## Results

*TRIM16 is down-regulated in GC while β-catenin, Cyclin D, and BCL2 are upregulated*

**Figure 1:**
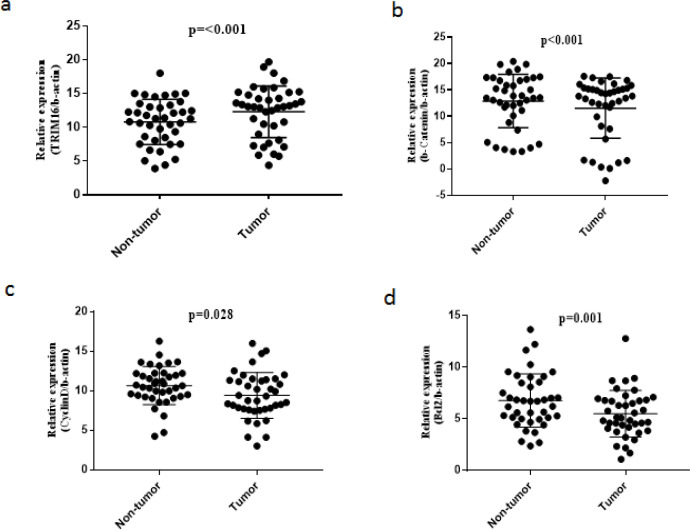
Gene Expression Analysis for TRIM16 (a), β-catenin (b), CyclinD (c) and BCL2 (d) in GC Tissues and Their Matched Noncancerous Controls

The results obtained from the preliminary analysis of qPCR are presented in Figure 1. In contrast to β-catenin, CyclinD and BCL2, the expression of TRIM16 is reduced in GC tissues. To clarify the meaning of Figure. 1, it should be noted that the value of expression level is correlated negatively with the number of PCR cycles; for example the lower Ct value represents a higher expression level for interested gene (Winer et al., 1999). 

According to Figure 1a, TRIM16 was down-regulated in 24 of 40 (60%) GC tissues (P<0.001) compared to the corresponding controls. β-catenin was significantly up-regulated in 30 (out of 40) GC samples (75%, P<0.001) (Figure 1b). Cyclin D was also overexpressed in 32 tissues (80%) of GC samples (P=0.028) (Figure 1c). At last, BCL2 expression was increased remarkably in 32 (out of 40) cancerous tissues (80%, P=0.001) (Figure 1d). The fold change (expression ratio) was calculated to compare the expression levels of the interested genes in GC tissues and controls (Table 2). Except TRIM16, other genes were up-regulated; β-Catenin, 2.53; Cyclin D, 2.36; and BCL2, 2.42.

*The correlation between TRIM16 expression and clinicopathological variables in GC patients*

Based on the TRIM16 expression, GC patients were divided in two groups (low and high) to study TRIM16 effects on clinicopathological variables (Table 3). The correlation between TRIM16 expression and the clinicopathological variables was not significant. 

*TRIM16 expression and the survival of GC patients*

**Figure 2:**
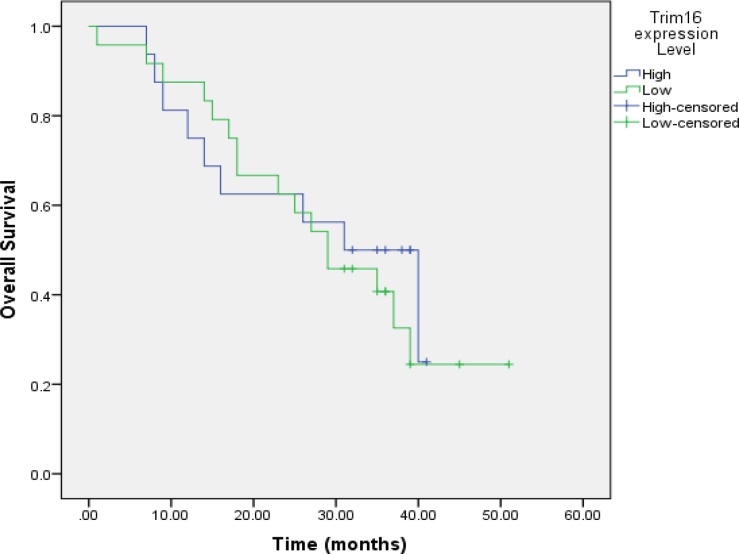
The Kaplan–Meier Survival Curve for Patients with GC Followed Up after Surgical Resection

During the survival time analysis for GC patients with high levels of TRIM16 expression, the mean and median survival time were estimated to be 27.938 and 31 months, respectively. Also, for patients with low levels of TRIM16 expression, the estimation of mean and median was performed (30.108 and 29, respectively). However, the log-rank (Mantel-Cox) test did not support the differences in mean and median estimations between the two groups (P=0.653). As it is illustrated in Figure 2, the trends of Kaplan–Meier survival curve are not significantly different for both of the groups.

The univariate and multivariate analyses were performed using Cox proportional hazard model to identify the factors associated with the overall survival in GC patients. Multivariate analysis was applied, independently, for those variables that were significant in the univariate analysis. Analysis by univariate Cox regression showed that anemia, weight loss, bleeding, stomach ache, and smoking were significantly linked to overall survival (Table 4). However, multivariate Cox regression analysis did not support the meaningful association of variables with overall survival (Table 5). 

**Table 1:** The primers’ Sequences that are Used in qPCR

Gene	Forward primer	Reverse primer	Length
*β-actin*	5'-CACGAAACTACCTTCAACTCC-3'	5'-CATACTCCTGCTTGCTGATC-3'	265
*TRIM16*	5'-AGACTTGGAGCGGAAACTCA-3'	5'-CTCACAGCAGCAAGGAGTTC-3'	138
*CyclinD*	5'-TGGAGGTCTGCGAGGAACA-3'	5'-TCATCTTAGAGGCCACGAACAT-3'	147
*BCL2 *	5'-ACGGTGGTGGAGGAGCTCTT-3'	5'-CGGTTGACGCTCTCCACAC-3'	98
*β-catenin*	5'-TCTGAGGACAAGCCACAAGATTACA-3'	5'-TGGGCACCAATATCAAGTCCAA-3'	122

**Table 2.** The Expression Ratio of Targeted Genes in GC Tissues

Gene	Fold Change (2^-^^ΔΔ^^ct^)	Expression Status
*TRIM16*	0.351	Down-expression
*β-Catenin*	2.53	Up expression
*Cyclin D*	2.36	Up expression
*BCL2*	2.42	Up expression

**Table 3:** Correlation between TRIM16 Level and Clinicopathological Features in GC Patients

Variables		TRIM16 Expression		P-value
		Low (n=24)	High (n=16)	
Age (mean ± sd)*	-	65.58 ± 9.48	65.93 ± 11.57	0.916
Age Composition**	≤65	14 (58.3)	7 (43.8)	0.366
	>65	10 (41.7)	9 (56.3)	
Gender**	Male (%)	19 (79.2)	12 (75.0)	0.525
	Female (%)	5 (20.8)	4 (25.0)	
Anemia**	+	17 (70.8)	10 (62.5)	0.581
	-	7 (29.2)	6 (37.5)	
Weight loss**	+	19 (79.2)	13 (81.3)	0.872
	-	5 (20.8)	3 (18.8)	
Dysphagia***	No	2 (8.3)	1 (6.3)	0.242
	Rarely	7 (29.2)	2 (12.5)	
	Yes	15 (62.5)	13 (0.81)	
	Proximal	10 (47.6)	8 (50.0)	0.885
Tumor location***	Body	8 (38.1)	5 (31.3)	
	Distal	3 (16.2)	3 (18.8)	
Smoking**	Yes	12 (50.0)	6 (37.5)	0.436
	No	12 (50.0)	10 (62.5)	
Stomach ache**	Yes	18 (75.0)	11 (68.8)	0.665
	No	6 (25.0)	5 (31.3)	
Nausea**	Yes	16 (66.7)	12 (75.0)	0.573
	No	8 (33.3)	4 (25.0)	
Reflux**	Yes	12 (50.0)	9 (56.3)	0.698
	No	12 (50.0)	7 (43.8)	
Anorexia**	Yes	8 (33.3)	7 (43.8)	0.505
	No	16 (66.7)	9 (56.3)	

T-test (*); Chi-square test (**); Fisher's exact test (***) were used to compare two groups.

**Table 4:** Univariate Analysis for Variables Associated with the Overall Survival of GC Patients

Variables	Univariate analysis		
	HR	95 % CI	P-value
TRIM16	1.02	0.81-1.29	0.887
Age	1.04	1.00-1.09	0.069
Gender	0.34	0.10-1.15	0.082
Tumor location	1.6	0.87-2.95	0.132
Anemia	0.12	0.00-0.38	0.014
Weight loss	0.26	0.00-0.98	0.049
Bleeding	0.28	0.11-0.75	0.011
Dysphagia	0.81	0.602-1.10	0.177
Stomach ache	0.24	0.07-0.80	0.02
Smoking	0.38	0.162-0.88	0.023
Nausea and Vomiting	0.41	0.151-1.10	0.076
Reflux	1.28	0.578-2.82	0.546
Anorexia	0.51	0.224-1.17	0.112

**Table 5:** Multivariate Analyses for Variables Associated with the Overall Survival of GC Patients

Variables	Multivariate analysis		
	HR	95 % CI	P-value
TRIM16	1.36	0.83-2.23	0.29
Anemia	0.001	0.00-3.34	0.93
Weight loss	0.001	0.00-5.86	0.945
Bleeding	0.586	0.21-1.67	0.32
Stomach ache	0.705	0.18-2.78	0.619

## Discussion

There is evidence that some members of the TRIM family play important roles in different cancers (Meroni, 2012). It has been demonstrated that TRIM16 plays a significant role in the retinoid signaling pathway and makes retinoid-resistant cancerous cells vulnerable to retinoic acid (Cheung et al., 2006). In neuroblastoma, the overexpression of TRIM16 reduces cell growth, cell motility and tumourigenicity (Marshall et al., 2010). The expression level of TRIM16 in prostate cancer was decreased and associated with patients’ overall survival (Qi et al., 2016). Other studies on prostate carcinoma and NSCLC, have shown that higher levels of TRIM16 lead to the inhibition of the EMT process and tumor cells invasion (Huo et al., 2015; Qi et al., 2016). In breast cancer and neuroblastoma cells, it has been confirmed that TRIM16 overexpression induces apoptosis through the induction of caspase-2 activity (Kim et al., 2013). Therefore, questions have been raised about the exact role of TRIM16, suppressor or activator, in different cancers. 

Previous published studies are limited and unsatisfactory about the role of TRIM16 in gastric cancer. Yan et al. claimed that TRIM16 level is increased in stage IV of GC tissues and in distant metastasis sites (Yan et al., 2015). This study suggested a positive correlation between TRIM16 expression and GC progression. Indeed, using RNAi, they showed that the inhibition of TRIM16 reduced the invasiveness of GC in vitro. Moreover, this research suggested TRIM16 as a potential target for SDMGC (lncRNA special for distant metastasis of GC; which is located on chromosome 17). A positive correlation between SDMGC expression and TRIM16 level is reported. In fact, they demonstrated that the up-regulation of SDMGC or TRIM16 increases cell invasion and migration in GC cell lines, while down-regulation works in the opposite direction.

 Here in the present study we tried to evaluate TRIM16 functions in Iranian GC patients. To do this, we recruited 40 patients with GC and obtained GC tissues and their corresponding adjacent noncancerous tissues (located at 5cm away from cancerous sites) by endoscopy. Using qPCR, we compared mRNA levels for TRIM16, β-catenin, CyclinD, BCL2 between cancerous and noncancerous tissues. Moreover, we analyzed the role of TRIM16 in patients’ overall survival and its association with patients’ clinicopathological profile. 

Yan et al., (2015) reported the overexpression of TRIM16 mRNA in distant metastasis samples, compared to stage IV cancer specimens, and higher levels of TRIM16 mRNA in stage IV cancer tissues in comparison with non-stage IV cancer tissues. However, in the present study we observed reduction in TRIM16 expression in GC tissues. This discrepancy could be attributed to study design, cancer stage, number of candidates and even the ethnic background. These results indicate that no single study could cover all aspects of TRIM16 in GC and further investigations are mandatory to define its contribution precisely. 

Comparing the overall survival between GC patients with higher levels of TRIM16 and those with lower TRIM16 expression, we found no meaningful link between the expression level of TRIM16 and overall survival. The Kaplan–Meier survival curve (Figure 2) also supported this observation. In addition to this, we did not discover any significant correlation between TRIM16 expression and clinicopathological variables (Table 3). Univariate Cox regression analysis (Table 4) indicated that anemia, weight loss, bleeding, stomach ache and smoking are statistically associated with overall survival, while multivariate analysis did not demonstrate any correlation between the variables and overall survival (Table 5). 

Here in this study we also measured the relative expressions of β-catenin, CyclinD and BCL2 genes. Previously, the activation of Wnt/β-catenin signaling pathway has been observed in 30-50% of GC samples (Chiurillo, 2015). Besides, it has been suggested that the overexpression of CyclinD is associated with GC progression and low survival rates (Takano et al., 1999; Yu et al., 2001; Shan et al., 2017). Furthermore, the expression level of BCL2 in GC has been the subject of a number of studies indicating its potential role(s) in GC formation and development (Saegusa et al., 1996; Van der Woude et al., 2003; Smith et al., 2005; Tsamandas et al., 2009; Yildirim et al., 2012; Gryko et al., 2014). In the current study, using qPCR, we figured out up-expression in fold changes for β-catenin (2.53), CyclinD (2.36) and BCL2 (2.42) genes. This result accords with previous studies, which showed that these genes are engaged with GC tumor progression. There was, however, a reduction in TRIM16 expression levels in GC tissues. A rational explanation for these observations is that TRIM16 plays a tumor suppressive role in GC and hindering its expression could result in higher expression levels for these three tumorigenic genes (β-catenin, CyclinD and BCL2). Hence, it could conceivably be hypothesized that there is a direct link between TRIM16 down-regulation and GC progression. However, further research should be undertaken to investigate the precise function(s) of TRIM16 in GC. 

In conclusion, this study was undertaken to examine TRIM16 expression patterns in Iranian GC patients and to investigate its possible association with patients’ overall survival. This research has identified lower expression levels of TRIM16 mRNA in GC tissues in comparison to their corresponding adjacent noncancerous samples. However, we found no significant correlation between TRIM16 expression and overall survival. Moreover, the evidence from this study suggests that reduction in TRIM16 mRNA level results in overexpression of the tumorigenic genes of β-catenin, CyclinD and BCL2. It can therefore be assumed that TRIM16 plays a tumor suppressive role in GC. Although further work is required to establish the validity of such a role for TRIM16, these findings help us to better understand the involved molecular pathways in GC formation and progression. Furthermore, the results of this study are encouraging for future studies on TRIM16 as a prognostic biomarker and potential target for GC management.

## Author Contribution Statement

Study concept and design: Mehrad-Majd H, Mehrzad J. Acquisition of data: Afshar J, Goshayeshi L. Statistical analysis: Mehrad-Majd H, Afshar J. Drafting of the manuscript: Mehrad-Majd H, Afshar J, Saeidi J. Study supervision: Mehrad-Majd H
